# The Efficacy of Interdisciplinary Near-Peer Teaching Within Neuroanatomical Education—Preliminary Observations

**DOI:** 10.1007/s40670-021-01238-6

**Published:** 2021-02-19

**Authors:** Charles F. C. Taylor, Octavia R. Kurn, Steven P. Glautier, Deepika Anbu, Oliver Dean, Eva Nagy, Kate R. Geoghan, Charlie H. Harrison, December R. Payne, Sam Hall, Scott Border

**Affiliations:** 1 Centre for Learning Anatomical Sciences, Faculty of Medicine, University of Southampton, Southampton, UK; 2 Department of Psychology, Faculty of Environment and Life Sciences, University of Southampton, Southampton, UK

**Keywords:** Neuroanatomy, Anatomy education, Near-peer teaching, Peer-assisted learning, Interdisciplinary education

## Abstract

Near-Peer Teaching (NPT) is increasingly becoming an integral part of the medical curriculum. When considered alongside the increasing popularity of interdisciplinary education, it seems appropriate to explore NPT within an interdisciplinary context. In these observations, 3rd-year medical students taught 2nd-year psychology students neuroanatomy. The session was evaluated using three objective and subjective assays. A knowledge assessment test showed significant improvement after teaching, and there were significant improvements on self-perceived knowledge/attitudes towards neuroanatomy, as well as positive feedback on the use of NPT. These observations evidence the successful use of Interdisciplinary Near-Peer Teaching (INPT) within a neuroanatomical curriculum.

**Supplementary Information:**

The online version contains supplementary material available at 10.1007/s40670-021-01238-6.

## Background

The concept of Near-Peer Teaching (NPT) as a form of Peer-Assisted Learning (PAL) [1] was first introduced in the late 1980s by Whitman [2]. Since then, a growing amount of pedagogical evidence for the effectiveness of NPT has been established to support its implementation within medical and anatomical curricula [3–14]. Despite there being some degree of discrepancy in how best to define NPT, it can be broadly described as teaching from ‘a student more advanced on the same curriculum’ [15]. In the instance of Interdisciplinary NPT/PAL, the definition is suggested only to be applicable when near-peers studying in different disciplines are at the same academic year level [1]; however, some studies have challenged this definition [16].

Given the expectation of our future healthcare professionals to serve as both practitioners and educators [17], NPT appears to not only be a beneficial curricular consideration but perhaps an essential one [18, 19]. If sufficient opportunities to develop teaching experiences do not exist within a student’s own programme, it is sensible to develop them elsewhere, especially where there is sufficient overlap with other subject areas.

However, there are factors that might limit how the NPT model could be successfully transferred between disciplines. One vital component considered to be responsible for the educational efficacy of NPT comes through the published literature on cognitive and social congruence [20, 21]. Cognitive congruence refers to the sharing of a familiar knowledge framework and includes the ability to use one’s own learning experiences to predict problems that students will likely encounter [21, 22]. Social congruence refers to the sharing of similar interpersonal attributes which enable improved communication within teaching scenarios [21, 23]. Ensuring a good proximity of cognitive and social congruence between disciplines may impact the success of interdisciplinary NPT (INPT). Within an interdisciplinary context, it is reasonable to assume that Near-Peer Teachers will have a less intuitive understanding of the learners’ curriculum and as a consequence may be less effective as role models.

A further potential barrier to the success of INPT originates from judging the educational distance between the near-peers. Existing knowledge on optimizing the educational gap between teacher and learner has originated from research conducted from studies using the same educational pathway [24, 25]. Current conclusions have suggested that teachers who are too far removed from the undergraduate experiences of the students may fail to share congruencies and that this impacts on their rapport [24, 25]. It therefore becomes difficult to meaningfully compare stages of training and degrees of congruency between Near-Peer Teachers on standard UK medical programs with standard non-medical classified degrees in the UK [24, 25].

Small increases in distance along the near-peer teaching spectrum have an impact upon the student’s perception of their learning experience, and therefore, there may be concern that INPT would not be successful. Consequently, it seems appropriate to explore whether these pedagogical concerns are indeed valid limiting factors for INPT. The current observations evaluate an INPT workshop to try and achieve this. In this practical neuroanatomy INPT exercise, undergraduate medical students were ‘Near-Peer Teachers’ of psychology undergraduates. These two disciplines were felt to be appropriate since there was a sufficient level of overlap in the learning requirements of their students.

## Activity

### The Near-Peer Teaching Network

The Faculties anatomy NPT programme and peer teaching community, known as the NPT network, has been in operation since 2010 and was originally developed by students and staff to support clinical neuroanatomy knowledge for clinical year students on placement. It is now an established and integral aspect of curriculum teaching within the pre-clinical phase of the programme. The network is an innovative partnership model between anatomy staff and students devised by the anatomy module lead which involves over 8 h of training to prepare self-selecting students for curriculum teaching.

The NPT training process involves opportunities for micro teaching (with feedback), practical demonstration practice, and a session which covers educational theory which is jointly run by the Junior Association of Medical Educators. Although all students are eligible to volunteer to be part of the network, students must demonstrate a commitment to the training and committee meetings to be eligible for selection, as well as showing evidence of responding positively to constructive feedback during the training process. The selection panel consists of senior medical students and the anatomy module lead for neuroanatomy [26].

### Teachers, Participants, and Session Structure

Forty-two second-year psychology students attended a 2-h neuroanatomy workshop setup and delivered by third-year BM5 BM/BS undergraduate medical students. All NPTs had a 2-year experience in clinically applied anatomy (approximating between 7 and 10 h of neuroanatomy teaching experience per student). All selected students had also previously attended at least one 2-day NPT training course run by The Faculty. Student teachers were selected based on their interest for the subject and not on their academic attainment. However, it is very likely that those who enjoy learning neuroanatomy are those likely to perform better in assessments.

The student learners (psychology students) received an introductory group teaching session designed and delivered by the NPTs based on learning outcomes specified by the module leader. The psychology students rotated through 4 cadaver-based practical stations which were each led by an NPT. The teaching at each station begun with a 7-min introductory demonstration followed by a 10-min NPT-led exploration of the specimens. The learning outcomes were specified by staff, but the structure of delivery was devised by the NPT’s. Faculty members provided NPTs with relevant learning resources so that NPTs could utilise them to guide the planning of their session. The 4 stations were themed as follows:

- 1) Brainstem and cerebellum
- 2) Limbic system
- 3) Basal ganglia
- 4) Visual pathways and cortex

### Data Collection and Statistical Analysis

Student learners completed questions online using polling software VEVOX (Meetoo Limited Auga Technologies Ltd. Version 1.440).

1. Six one best answer (OBA) multiple choice questions (MCQs), each with 5 possible options, were used to assess the knowledge-based learning outcomes of the session. The same questions were used pre- and post-session, and results were compared using a Wilcoxon signed-rank test.
2. Self-perceived levels of neuroanatomical knowledge and attitudes towards neuroanatomy were assessed pre- and post-session with four 9-point Likert scale items with poles labelled ‘excellent’ or ‘strongly agree’ and ‘very poor’ or ‘strongly disagree’. The four statements used to assess the self-perceived levels of neuroanatomical knowledge were as follows:
  - I feel now that my level of knowledge in neuroanatomy is…
  - My confidence in neuroanatomy is currently…
  - I am anxious about learning neuroanatomy in the lab …
  - I am excited about learning neuroanatomy in the lab…
    Responses were compared pre- and post-session using a series of Wilcoxon signed rank tests. Self-perceived levels of neuroanatomical knowledge and attitudes towards neuroanatomy were also assessed with four additional post-session questionnaire items using 9-point Likert scales with poles labelled ‘strongly disagree’ and ‘strongly agree’. A series of one-sample Wilcoxon signed rank tests were used to compare responses to the scale mid-points.
3. Student evaluation of NPT was assessed with a single pre- and post-session item and with eight post session items. These used the same scales described for the pre- and post-self-perceived knowledge and attitude items described.
  All instruments are found in Online Resource 1.

## Results

1. Knowledge
  MCQ Pre-teaching mean knowledge scores were 53% correct (SD = 0.499), post-teaching scores were 71% correct (SD = 0.456). Wilcoxon signed-rank test indicated that the post-teaching scores were statistically significantly higher (*Z* = 4.781, *p* < 0.005). Thirty-one out of the 41 students who responded to the quiz (76%) had higher scores on the post-teaching test.
  When accounting for the high pre-test scores we found that the average normalised knowledge gain, (< g >  = (< Post >—< Pre >)/(100—< Pre >))[27] per student was + 49.20%
2. Self-perceived knowledge and attitudes.

Pre- and post-session assessments of all 42 students:

- Students’ perceived level of knowledge was significantly higher after the session than before (*Z* = 4.731, *p* < 0.005)—a mean raw score increase of 1.36.
- Student learners’ confidence in neuroanatomy was significantly higher after the session than before (*Z* = 4.977, *p* < 0.005)—a mean raw score increase of 2.15.
- Student learners felt significantly less anxious about learning anatomy in the lab after the session than before (Z = 2.633, *p* < 0.008)—a mean raw score decrease of 1.3.
- Student learners were significantly more excited about learning neuroanatomy in the lab after the session than before (*Z* = 2.008, *p* < 0.045), with a mean raw score increase of 0.55.
  Post session assessment
  Table 1 shows that for all four self-perceived knowledge and attitude items, the majority of all 42 student responses agreed with the statements indicating improvements after the session.

**Table 1:** Self-perceived knowledge and attitudes towards neuroanatomy and evaluations of NPT post session questions. Wilcoxon test against the Likert-scale midpoint, 5, neither agree nor disagree Student Learner evaluation of NPT and self-perceived knowledge gain

Question	Average response	One-sample Wilcoxon test result
This anatomy session has made me more interested in neuroanatomy?	7.85	(*Z* = 5.392, *p* < 0.005)
This anatomy session has made me more interested in my module (PSYC2025)?	7.56	(*Z* = 5.458, *p* < 0.005)
Using real human brains to teach neuroanatomy has enhanced my engagement with the topic?	8.29	(*Z* = 5.787, *p* < 0.005)
Using real human brains to teach neuroanatomy has enhanced my understanding of the topic?	8.17	(*Z* = 5.707, *p* < 0.005)
I would like to see peer teaching programmes set up within my own discipline?	7.23	(*Z* = 5.139, *p* < 0.005)
I preferred being taught by the students compared with faculty staff?	5.88	(*Z* = 2.480, *p* = 0.013)
I thought that the medical student teachers were approachable?	8.65	(*Z* = 5.817, *p* < 0.005)
I thought that the medical student teachers were knowledgeable in the areas I needed to know about?	8.49	(*Z* = 5.762, *p* < 0.005)
I thought that teaching by the students was clear?	8.46	(*Z* = 5.787, *p* < 0.005)
Being taught by students made the learning more enjoyable	7.28	(*Z* = 5.032, *p* < 0.005)
I thought that the student teachers had a unique way of conveying information?	8.32	(*Z* = 5.641, *p* < 0.005)
The experience of being taught by other students at the University has made me more interested in having a go at teaching others myself?	6.53	(*Z* = 3.909, *p* < 0.005)

3) Student evaluation of NPT
  Pre-and post-session attitudes:
  There was a significant difference in the pre- and post-survey responses between the number of student learners who thought that medical student teachers leading the session made it more useful (*Z* = 3.812, *p* < 0.005) with a mean raw score increase of 1.21. All students responded to the pre- and post-session evaluation.
  Post session assessment:
  Table 1 shows that for all eight of the post-session items, most of the 42 responses agreed with items indicating positive evaluations of NPT.
  Perceived vs actual knowledge gain:
  A comparison between perceived and actual knowledge gain showed a 9.68% greater increase in perceived knowledge gain.
  General student approach to neuroanatomy questionnaire—qualitative feedback:
  Qualitative feedback regarding the NPT aspect of the teaching session is shown in Table 2. Of the 27 responses, all were overwhelmingly positive and common themes included the perception that the NPTs were knowledgeable, were well informed and explained the content clearly. Several students also reported that the NPTs created a more approachable and less formal learning environment.
  All source data are found in Online Resource 2.

**Table 2:** Table showing the student learners’ feedback regarding their general approach to neuroanatomy. General student approach to neuroanatomy questionnaire—qualitative feedback

Please give reasons for why you did/did not like the peer teaching aspect of this session?
The peer teachers made me feel more confident to ask/answer questions because I didn’t mind being wrong or asking multiple times. Which made it a more valuable learning experience and more enjoyable
It was great, but I think we could have got more out of it if we are taught by those who know more about what is relevant for us/ what we already know (even if they know the brain less well)
Most of them were incredibly helpful and well informed
I just think that if we would have more pictures with the brain and the teaching would follow a clear structure about the brain parts it would be easier to visualise and remember info
Students were friendly and described things in ways that made it initially memorable for them
They were able to relate to students
They were really knowledgeable and friendly
The student teachers were very approachable and engaging
Friendly, knowledgeable
They were very approachable and friendly
Felt more approachable and relatable
They explained things at a level I could understand and retain information. More approachable and easy to relate to
They explain very clearly
It was less formal
It is more connected to topic. So, I would like to have sessions like that. Maybe even some VR teaching in the future
It was more engaging and less formal
Really great! Has fuelled my interest!
Possibly my fondest learning experience during my 3 years at University! So unique
More approachable, easier to ask questions
I liked it as they were more relatable and approachable and knew how to describe things simply which often lecturers don't
They were more approachable and had ways of teaching so we can remember things, but did not know what was relevant
More interactive
Good understanding of what some students typically face when given new stuff to learn
They made me feel not dumb asking questions. Thanks!
I really enjoyed it as they engaged everyone and made everything sound very clear—understood everything a lot better
They taught the topics in a very engaging way and made it much more understandable
The students made the course thoroughly enjoyable and thought provoking. They were friendly and talkative and lovely to learn from

## Discussion

The application of NPT as a form of peer-assisted learning [1] is used throughout higher education, and there has become a growing need for appropriate pedagogy to underpin its practice. Although the definition of a Near-Peer is generally well accepted [1], there is far less clarity when it comes to interdisciplinary programs. Despite numerous examples of interdisciplinary education occurring within anatomy and literature [10, 11, 13, 14, 28], there are very few publications on the interdisciplinary application of peer-assisted learning and any of the benefits it may bring.

Teaching experience and professional behavior can often be viewed as a critical skill to develop for future clinicians [5, 20, 29], and so medical students commonly seek out such opportunities [30, 31]. Therefore, interdisciplinary experience clearly affords advantages to both parties. However, congruence factors such as the use of familiar language and the sharing of similar social roles are thought to be critical for NPT success. Therefore, it is of interest to explore how they might differ amongst interdisciplinary examples and assess how sensitive they might be to misalignment.

Although medical students have no prior understanding of the curriculum strategies or assessment models of their psychology counterparts, the outcomes from our perceptions survey were remarkably similar to results that have been reported for single disciplinary programs in anatomy [25, 32, 33]. In this investigation, not being able to act directly as a translatable role model did not have a major detrimental impact on teacher/learner dynamics. Perhaps this is unsurprising as medical students have long been taught anatomy by faculty members and anatomy graduates with minimal translatable role modelling for future clinicians [34–36]. Despite the cross-program nature of the teaching, student learners still successfully acquired knowledge from the teaching session which is demonstrated through the calculation of both absolute and normalised gain.

Despite educational distance not being directly comparable, it is likely that congruence was negatively impacted upon to some degree by program differences. However, this did not appear to prevent learning gain or a positive teaching experience. This observation adds further weight to the more recent evidence suggesting the flexibility and versatility of NPT applications within undergraduate anatomy education [6, 25, 33].

In the current observations, our pre- and post-test comparison shows significant differences in the student’s perceived levels of neuroanatomical knowledge and confidence in learning neuroanatomy. The experience seemed to decrease anxiety and increased excitement towards learning neuroanatomy.

There can often be issues with students’ perceived levels of knowledge vs true knowledge gain as evidenced by objective outcome measures [32, 37]. However, it is useful to know if such an enjoyable and novel learning experience offers genuine knowledge gain or if this gain is due to the ‘wow’ factor in a new experience.

This teaching session resulted in a significantly greater proportion of students believing that near-peer teachers made the session more useful than if it had been delivered exclusively by the faculty, and the student learners agreed they would like to see peer-teaching programs in their own discipline. This is an encouraging sign that the experience was positive enough for them to welcome the further development of such schemes. The major qualitative feedback themes focused on social and cognitive congruence aspects, referring to the NPTs as creating a less formal, more engaging and approachable learning environment, where they felt more confident and able to ask questions. This is consistent with current research themes particularly within the context of medical education [38]. Our results are comparable to the language used when NPT has been deployed within anatomy education [10], and suggests that the benefits of congruence still exist in the interdisciplinary model.

### Limitations

The reliability of these observations are limited by the study’s small sample size and by the absence of a control group. It is therefore not possible to know if our observations are truly a result of the INPT workshop. Whilst this exploratory study goes some way to display a correlation between INPT and a successful teaching environment, it is unable to exclude the influence of both the structure and novelty of the session on the outcome. The absence of a control means that a causal relationship could not be established. However, it is a challenge to ethically run control groups in studies that involve a live curriculum as it may disadvantage students. This study takes a tentative first step in suggesting that this approach is worth exploring.

Furthermore, the learning observations rely on a limited objective outcome measure based on immediate knowledge gain and cannot be considered a fully accurate representation of deeper knowledge acquisition that is retained over time. It would be of interest to carry out a further study using a more comprehensive knowledge assessment with a retention test using a moderate and extended test interval.

## Conclusion

In conclusion, these preliminary observations provide some tentative evidence for the potential to successfully integrate Interdisciplinary Near-Peer Teachers (INPTs) within the neuroanatomical curricula of a psychology program. However, the increased levels of knowledge and improved attitudes of the students are to be expected of pupils attending any successful learning session.

It would appear that the critical factors unique for NPT success, such as congruence factors and educational distance, are adaptable when the a INPT model is adopted. We would however encourage further, more robust studies to explore this in greater detail.

## Supplementary Information

Below is the link to the electronic supplementary material.

Supplementary file1 (DOCX 98 KB)

Supplementary file2 (DOCX 38 KB)

## Data Availability

The datasets generated during and/or analysed during the current study are available from the corresponding author on reasonable request and were provided on original submission of the article as additional supporting files.
