# Supplementary material for: The Efficacy of Interdisciplinary Near-Peer Teaching Within Neuroanatomical Education—Preliminary Observations
Source: Med Sci Educ. 2021 Feb 19;31(2):387–93. doi: 10.1007/s40670-021-01238-6 (PMC8368458; doi:10.1007/s40670-021-01238-6)
Supplement: Supplementary file 1 — Supplementary file1 (DOCX 98 KB) [file 40670_2021_1238_MOESM1_ESM.docx]

Instruments:

# 1: Pre- and Post-session VEVOX knowledge assessment

**Question 1: The function of Broca’s area is…**

Choice: Description

1 Processing and understanding of speech

2 Forming memories

3 Recognising faces

4 Production of speech

5 Interpreting visual information

**Question 2: The cerebral aqueduct provides a route for passage of CSF between…**

Choice: Description

1 The first and second ventricle

2 The lateral ventricles and the third ventricle

3 The third and fourth ventricles

4 The fourth ventricle and the spinal cord

5 The two lateral ventricles

**Question 3: The area of the brain associated with recognising faces is the…**

Choice: Description

1 Occipital lobe

2 Temporal lobe

3 Broca’s Area

4 Wernicke’s area

5 Fusiform gyrus

**Question 4: The function of the fornix is…**

Choice: Description

1 Output tract from the hippocampus

2 Consolidation of short-term memories to long-term

3 Relay station of the brain

4 Centre for emotions relating to survival

5 Regulation of body temperature

**Question 5: The substantia nigra contains…. Neurons**

Choice: Description

1 Noradrenergic

2 Adrenergic

3 Serotoninergic

4 Dopaminergic

5 Cholinergic

**Question 6: The primary visual cortex is located in the… lobe of the brain**

Choice: Description

1 Frontal

2 Occipital

3 Temporal

4 Parietal

5 Cerebellum

|  | Before the session | | | | | | | | |
| --- | --- | --- | --- | --- | --- | --- | --- | --- | --- |
|  | **very poor** |  | **poor** |  | **average** |  | **good** |  | **excellent** |
| I feel that my current level of knowledge in neuroanatomy is… |  |  |  |  |  |  |  |  |  |
| My confidence in neuroanatomy is currently… |  |  |  |  |  |  |  |  |  |
|  | **strongly disagree** |  | **disagree** |  | **neither agree nor disagree** |  | **agree** |  | **strongly agree** |
| I am anxious about learning neuroanatomy in the lab |  |  |  |  |  |  |  |  |  |
| I am excited about learning neuroanatomy in the lab |  |  |  |  |  |  |  |  |  |
|  |  |  |  |  |  |  |  |  |  |
|  | After the session | | | | | | | | |
|  | **very poor** |  | **poor** |  | **average** |  | **good** |  | **excellent** |
| I feel that my current level of knowledge in neuroanatomy is… |  |  |  |  |  |  |  |  |  |
| My confidence in neuroanatomy is currently… |  |  |  |  |  |  |  |  |  |
|  | **strongly disagree** |  | **disagree** |  | **neither agree nor disagree** |  | **agree** |  | **strongly agree** |
| I am anxious about learning neuroanatomy in the lab |  |  |  |  |  |  |  |  |  |
| I am excited about learning neuroanatomy in the lab |  |  |  |  |  |  |  |  |  |

|  | Post-Session | | | | | | | | |
| --- | --- | --- | --- | --- | --- | --- | --- | --- | --- |
|  | **strongly disagree** |  | **disagree** |  | **neither agree nor disagree** |  | **agree** |  | **strongly agree** |
| This anatomy session has made me more interested in neuroanatomy? |  |  |  |  |  |  |  |  |  |
| This anatomy session has made me more interested in my module (PSYC2025)? |  |  |  |  |  |  |  |  |  |
| Using real human brains to teach neuroanatomy has enhanced my engagement with the topic? |  |  |  |  |  |  |  |  |  |
| Using real human brains to teach neuroanatomy has enhanced my understanding of the topic? |  |  |  |  |  |  |  |  |  |

# 2: Self-perceived levels of neuroanatomical knowledge and attitudes towards neuroanatomy

|  | Before the session | | | | | | | | |
| --- | --- | --- | --- | --- | --- | --- | --- | --- | --- |
|  | **strongly disagree** |  | **disagree** |  | **neither agree nor disagree** |  | **agree** |  | **strongly agree** |
| I think that student teachers leading the session will make it more useful |  |  |  |  |  |  |  |  |  |
|  |  |  |  |  |  |  |  |  |  |
|  | After the session | | | | | | | | |
|  | **strongly disagree** |  | **disagree** |  | **neither agree nor disagree** |  | **agree** |  | **strongly agree** |
| I think that student teachers leading the session will make it more useful |  |  |  |  |  |  |  |  |  |

|  | After the session | | | | | | | | |
| --- | --- | --- | --- | --- | --- | --- | --- | --- | --- |
| I would like to see peer teaching programmes set up in my own discipline? | **strongly disagree** |  | **disagree** |  | **neither agree nor disagree** |  | **agree** |  | **strongly agree** |
| I preferred being taught by the students compared to faculty staff? |  |  |  |  |  |  |  |  |  |
| I thought that the medical student teachers were approachable? |  |  |  |  |  |  |  |  |  |
| I thought that the medical student teachers were knowledgeable in the areas I needed to know about? |  |  |  |  |  |  |  |  |  |
| The teaching by the students was clear? |  |  |  |  |  |  |  |  |  |
| Being taught by students made the learning more enjoyable |  |  |  |  |  |  |  |  |  |
| I thought that the student teachers had a unique way of conveying information? |  |  |  |  |  |  |  |  |  |
| The experience of being taught by other students at the University has made me more interested in having a go at teaching others myself? |  |  |  |  |  |  |  |  |  |

# 3: Student evaluation of NPT

Please give reasons for why you did/did not like the peer teaching aspect of this session?

# 4: Post-session qualitative feedback
