# Supplementary material for: The Efficacy of Interdisciplinary Near-Peer Teaching Within Neuroanatomical Education—Preliminary Observations
Source: Med Sci Educ. 2021 Feb 19;31(2):387–93. doi: 10.1007/s40670-021-01238-6 (PMC8368458; doi:10.1007/s40670-021-01238-6)
Supplement: Supplementary file 2 — Supplementary file2 (DOCX 38 KB) [file 40670_2021_1238_MOESM2_ESM.docx]

Pre-session feedback

| Respondent number | Respondent Gender | Student number | Question 1 | Question 2 | Question 3 | Question 4 | Question 5 |
| --- | --- | --- | --- | --- | --- | --- | --- |
| 1 | 2 | 29612624 | 5 | 3 | 5 | 9 | 5 |
| 2 | 2 | 28035534 | 2 | 1 | 1 | 9 | 5 |
| 3 | 2 | 29414903 | 5 | 5 | 1 | 9 | 7 |
| 4 | 2 | 28751487 | 3 | 3 | 6 | 7 | 6 |
| 5 | 2 | 29820251 | 5 | 5 | 2 | 9 | 7 |
| 6 | 1 | 29508959 | 5 | 5 | 5 | 7 | 5 |
| 7 | 2 | 29250838 | 5 | 4 | 9 | 6 | 7 |
| 8 | 1 | 29730838 | 4 | 3 | 7 | 6 | 7 |
| 9 | 2 | 29450845 | 5 | 4 | 5 | 8 | 8 |
| 10 | 2 | 29781469 | 5 | 3 | 7 | 7 | 8 |
| 11 | 2 | 29276144 | 6 | 5 | 2 | 9 | 8 |
| 12 | 2 | 29123046 | 5 | 4 | 5 | 7 | 6 |
| 13 | 2 | 29335213 | 6 | 5 | 1 | 9 | 7 |
| 14 | 2 | 27680428 | 6 | 6 | 1 | 8 | 7 |
| 15 | 2 | 28697936 | 5 | 3 | 3 | 9 | 7 |
| 16 | 1 | 29641616 | 5 | 3 | 3 | 8 | 8 |
| 17 | 1 | 29405491 | 5 | 5 | 3 | 8 | 7 |
| 18 | 2 | 29302161 | 4 | 4 | 1 | 9 | 9 |
| 19 | 2 | 29446368 | 4 | 4 | 3 | 7 | 7 |
| 20 | 2 | 27820343 | 6 | 5 | 4 | 8 | 9 |
| 21 | 2 | 27528642 | 5 | 5 | 3 | 7 | 7 |
| 22 | 2 | 29517699 | 5 | 4 | 2 | 6 | 7 |
| 23 | 2 | 29386853 | 2 | 2 | 5 | 6 | 6 |
| 24 | 2 | 29570301 | 5 | 4 | 2 | 9 | 9 |
| 25 | 2 | 29873312 | 6 | 5 | 4 | 9 | 7 |
| 26 | 2 | 28423283 | 2 | 2 | 5 | 6 | 6 |
| 27 | 1 | 29512549 | 3 | 3 | 1 | 7 | 5 |
| 28 | 2 | 29575621 | 3 | 3 | 5 | 7 | 5 |
| 29 | 2 | 29258863 | 5 | 4 | 6 | 8 | 8 |
| 30 | 2 | 29363993 | 3 | 3 | 4 | 7 | 7 |
| 31 | 2 | 29594847 | 3 | 3 | 5 | 7 | 7 |
| 32 | 2 | 29173134 | 3 | 3 | 5 | 7 | 7 |
| 33 | 2 | 29436467 | 4 | 3 | 2 | 2 | 7 |
| 34 | 1 | 26383773 | 6 | 6 | 2 | 9 | 8 |
| 35 | 2 | 27109151 | 5 | 1 | 1 | 9 | 7 |
| 36 | 2 | 29314461 | 6 | 5 | 7 | 8 | 4 |
| 37 | 2 | 29268834 | 5 | 4 | 3 | 9 | 5 |
| 38 | 2 | 29457602 | 5 | 5 | 3 | 8 | 7 |
| 39 | 2 | 30588138 | 3 | 3 | 1 | 9 | 9 |
| 40 | 2 | 29094836 | 4 | 4 | 1 | 9 | 7 |
| 41 | 2 | 29331846 | 3 | 3 | 1 | 7 | 7 |
| 42 | 1 | 29325307 | 3 | 3 | 3 | 8 | 7 |

Post- session feedback

| Respondent number | Respondent Gender | Student number | Question 1 | Question 2 | Question 3 | Question 4 | Question 5 |
| --- | --- | --- | --- | --- | --- | --- | --- |
| 1 | F | 29612624 | 6 | 6 | 2 | 9 | 8 |
| 2 | F | 28035534 | 1 | 2 | 1 | 8 | 3 |
| 3 | F | 29414903 | 7 | 6 | 1 | 9 | 7 |
| 4 | F | 28751487 | 5 | 6 | 8 | 9 | 9 |
| 5 | F | 29820251 | 6 | 6 | 1 | 9 | 8 |
| 6 | M | 29508959 | 7 | 6 | 1 | 9 | 9 |
| 7 | F | 29250838 |  |  |  |  |  |
| 8 | M | 29730838 | 7 | 8 | 3 | 9 | 9 |
| 9 | F | 29450845 |  |  |  |  |  |
| 10 | F | 29781469 |  |  |  |  |  |
| 11 | F | 29276144 | 7 | 7 | 3 | 8 | 8 |
| 12 | F | 29123046 | 6 | 6 | 1 | 8 | 8 |
| 13 | F | 29335213 |  |  |  |  |  |
| 14 | F | 27680428 | 7 | 7 | 1 | 8 | 9 |
| 15 | F | 28697936 | 6 | 6 | 3 | 7 | 7 |
| 16 | M | 29641616 | 7 | 8 | 3 | 9 | 9 |
| 17 | M | 29405491 | 7 | 6 | 2 | 8 | 9 |
| 18 | F | 29302161 |  |  |  |  |  |
| 19 | F | 29446368 | 7 | 7 | 2 | 7 | 7 |
| 20 | F | 27820343 |  |  |  |  |  |
| 21 | F | 27528642 | 6 | 6 | 3 | 7 | 7 |
| 22 | F | 29517699 | 5 | 7 | 1 | 9 | 9 |
| 23 | F | 29386853 | 3 | 3 | 3 | 7 | 7 |
| 24 | F | 29570301 | 5 | 4 | 2 | 9 | 9 |
| 25 | F | 29873312 |  |  |  |  |  |
| 26 | F | 28423283 |  |  |  |  |  |
| 27 | M | 29512549 | 7 | 8 | 3 | 9 | 9 |
| 28 | F | 29575621 | 3 | 3 | 3 | 7 | 7 |
| 29 | F | 29258863 | 6 | 6 | 4 | 7 | 7 |
| 30 | F | 29363993 | 4 | 4 | 1 | 7 | 7 |
| 31 | F | 29594847 | 5 | 7 | 1 | 9 | 9 |
| 32 | F | 29173134 | 5 | 5 | 1 | 9 | 9 |
| 33 | F | 29436467 | 6 | 5 | 2 | 9 | 9 |
| 34 | M | 26383773 | 8 | 8 | 1 | 9 | 9 |
| 35 | F | 27109151 | 5 | 5 | 1 | 9 | 9 |
| 36 | F | 29314461 | 7 | 7 | 1 | 9 | 9 |
| 37 | F | 29268834 | 8 | 7 | 2 | 9 | 8 |
| 38 | F | 29457602 | 7 | 7 | 1 | 9 | 9 |
| 39 | F | 30588138 | 5 | 7 | 1 | 9 | 9 |
| 40 | F | 29094836 | 6 | 6 | 1 | 9 | 9 |
| 41 | F | 29331846 | 5 | 5 | 6 | 5 | 5 |
| 42 | M | 29325307 | 4 | 4 | 3 | 7 | 9 |

Feedback

| Respondent number | Respondent Gender | Student number | Question 1 | Question 2 | Question 3 | Question 4 | Question 5 | Question 6 | Question 7 | Question 8 | Question 9 | Question 10 | Question 11 | Question 12 | Question 13 | Question 14 | Question 15 | Question 16 | Question 17 | Question 18 | Question 19 | Question 20 |
| --- | --- | --- | --- | --- | --- | --- | --- | --- | --- | --- | --- | --- | --- | --- | --- | --- | --- | --- | --- | --- | --- | --- |
| 1 | F | 29612624 | 9 | 9 | 9 | 9 | 9 | 9 | 8 | 8 | 7 | 9 | 8 | 9 | 9 | 9 | 8 | 7 | 7 | 5 | 7 | 0 |
| 2 | F | 28035534 | 5 | 4 | 6 | 7 | 7 | 3 | 7 | 6 | 6 | 5 | 3 | 8 | 8 | 7 | 5 | 5 | 3 | 3 | 3 | 1 |
| 3 | F | 29414903 | 7 | 9 | 9 | 9 | 9 | 9 | 7 | 7 | 5 | 5 | 6 | 9 | 9 | 9 | 7 | 5 | 5 | 3 | 5 | 0 |
| 4 | F | 28751487 | 8 | 9 | 9 | 9 | 9 | 9 | 9 | 9 | 9 | 9 | 9 | 9 | 9 | 9 | 9 | 9 | 9 | 8 | 9 | 1 |
| 5 | F | 29820251 | 7 | 8 | 8 | 9 | 9 | 7 | 6 | 7 | 5 | 9 | 8 | 9 | 5 | 6 | 8 | 4 | 6 | 4 | 6 | 1 |
| 6 | M | 29508959 | 9 | 9 | 9 | 9 | 9 | 7 | 6 | 7 | 7 | 7 | 8 | 9 | 8 | 9 | 9 | 7 | 7 | 5 | 7 | 1 |
| 7 | F | 29250838 | 7 | 9 | 9 | 9 | 8 | 8 | 4 | 8 | 8 | 8 | 8 | 9 | 9 | 9 | 9 | 7 | 6 | 5 | 7 | 1 |
| 8 | M | 29730838 | 8 | 8 | 9 | 9 | 9 | 9 | 9 | 9 | 9 | 8 | 8 | 8 | 8 | 9 | 8 | 9 | 9 | 9 | 9 | 1 |
| 9 | F | 29450845 | 8 | 9 | 9 | 9 | 9 | 8 | 9 | 8 | 5 | 8 | 8 | 9 | 9 | 9 | 9 | 7 | 6 | 6 | 8 | 1 |
| 10 | F | 29781469 | 7 | 7 | 7 | 8 | 8 | 6 | 8 | 8 | 6 | 7 | 7 | 9 | 9 | 9 | 9 | 9 | 3 | 8 | 8 | 1 |
| 11 | F | 29276144 | 7 | 8 | 7 | 7 | 8 | 8 | 7 | 7 | 6 | 5 | 7 | 9 | 8 | 8 | 9 | 7 | 4 | 1 | 7 | 1 |
| 12 | F | 29123046 | 7 | 8 | 9 | 9 | 9 | 9 | 9 | 9 | 8 | 8 | 8 | 9 | 9 | 9 | 9 | 7 | 8 | 8 | 8 | 1 |
| 13 | F | 29335213 | 9 | 9 | 9 | 9 | 9 | 9 | 8 | 9 | 5 | 9 | 9 | 9 | 9 | 9 | 9 | 9 | 9 | 9 | 9 | 1 |
| 14 | F | 27680428 | 9 | 9 | 9 | 9 | 9 | 9 | 3 | 9 | 9 | 9 | 9 | 9 | 9 | 9 | 9 | 9 | 9 | 5 | 5 | 1 |
| 15 | F | 28697936 | 9 | 9 | 9 | 9 | 9 | 9 | 9 | 9 | 9 |  |  | 0 |  |  |  | 8 | 6 | 8 |  | 1 |
| 16 | M | 29641616 | 7 | 9 | 9 | 9 | 9 | 9 | 7 | 8 | 9 | 8 | 8 | 9 | 9 | 7 | 8 | 9 | 9 | 7 | 8 | 1 |
| 17 | M | 29405491 | 9 | 9 | 7 | 9 | 8 | 7 | 7 | 7 | 6 | 7 | 7 | 9 | 8 | 9 | 8 | 5 | 5 | 2 | 6 | 1 |
| 18 | F | 29302161 | 9 | 9 | 9 | 9 | 9 | 9 | 6 | 9 | 6 | 9 | 6 | 9 | 9 | 9 | 9 | 9 | 9 | 9 | 9 | 1 |
| 19 | F | 29446368 | 7 | 7 | 7 | 7 | 8 | 7 | 5 | 7 | 6 | 8 | 8 | 8 | 8 | 8 | 8 | 7 | 7 | 7 | 7 | 1 |
| 20 | F | 27820343 | 9 | 9 | 9 | 9 | 9 | 7 | 5 | 9 | 9 | 9 | 9 | 9 | 9 | 9 | 8 | 9 | 6 | 3 | 9 | 1 |
| 21 | F | 27528642 | 7 | 9 | 9 | 9 | 9 | 7 | 6 | 7 | 5 | 8 | 7 | 9 | 8 | 8 | 7 | 7 | 5 | 6 | 7 | 1 |
| 22 | F | 29517699 | 7 | 7 | 8 | 9 | 9 | 8 | 5 | 7 | 6 | 7 | 6 | 9 | 9 | 9 | 8 | 7 | 6 | 5 | 7 | 1 |
| 23 | F | 29386853 | 6 | 6 | 6 | 6 | 6 | 6 | 6 | 6 | 6 | 7 | 7 | 7 | 7 | 7 | 7 | 7 | 7 | 7 | 7 | 1 |
| 24 | F | 29570301 | 7 | 8 | 8 | 9 | 7 | 7 | 1 | 6 | 5 | 8 | 8 | 9 | 9 | 8 | 8 | 7 | 5 | 5 | 9 | 1 |
| 25 | F | 29873312 | 8 | 9 | 9 | 9 | 9 | 9 | 8 | 8 | 6 | 9 | 9 | 9 | 9 | 9 | 9 | 8 | 8 | 8 | 8 | 1 |
| 26 | F | 28423283 | 7 | 8 | 7 | 8 | 7 | 7 | 7 | 8 | 7 | 7 | 7 | 8 | 9 | 9 | 8 |  |  |  |  |  |
| 27 | M | 29512549 | 6 | 7 | 7 | 7 | 7 | 4 | 5 | 7 | 5 | 7 | 7 | 0 | 9 | 9 | 9 | 7 | 3 | 5 | 5 |  |
| 28 | F | 29575621 | 9 | 9 | 9 | 9 | 9 | 9 | 9 | 9 | 8 | 9 | 9 | 9 | 9 | 9 | 9 | 8 | 8 | 9 | 9 | 1 |
| 29 | F | 29258863 | 4 | 7 | 7 | 9 | 8 | 6 | 6 | 9 | 5 | 9 | 6 | 9 | 8 | 8 | 9 | 5 | 5 | 3 | 5 | 1 |
| 30 | F | 29363993 | 6 | 8 | 8 | 8 | 8 | 7 | 6 | 6 | 5 | 7 | 6 | 8 | 9 | 9 | 8 | 6 | 4 | 5 | 6 | 1 |
| 31 | F | 29594847 | 7 | 9 | 9 | 9 | 9 | 9 | 3 | 9 | 9 | 7 | 7 | 7 | 6 | 7 | 7 |  |  |  |  |  |
| 32 | F | 29173134 | 7 | 9 | 9 | 9 | 7 | 7 | 5 | 7 | 7 | 7 | 7 | 7 | 9 | 9 | 9 | 7 | 7 | 5 | 7 | 1 |
| 33 | F | 29436467 | 7 | 9 | 9 | 7 | 7 | 6 | 7 | 7 | 7 | 9 | 7 | 9 | 9 | 9 | 9 | 7 | 7 | 5 | 7 | 1 |
| 34 | M | 26383773 | 7 | 9 | 8 | 9 | 9 | 9 | 4 | 7 | 9 | 9 | 9 | 9 | 9 | 9 | 8 | 8 | 7 | 7 | 7 | 1 |
| 35 | F | 27109151 | 9 | 9 | 9 | 9 | 9 | 9 | 5 | 9 | 9 | 9 | 9 | 9 | 9 | 7 | 9 | 9 | 9 | 5 | 9 | 1 |
| 36 | F | 29314461 | 7 | 7 | 8 | 9 | 7 | 6 | 5 | 8 | 5 | 9 | 9 | 9 | 9 | 8 | 8 | 5 | 6 | 5 | 7 | 1 |
| 37 | F | 29268834 | 7 | 7 | 8 | 8 | 8 | 8 | 6 | 7 | 7 | 8 | 8 | 8 | 8 | 8 | 8 | 5 | 7 | 5 | 6 | 1 |
| 38 | F | 29457602 | 7 | 9 | 9 | 9 | 9 | 8 | 5 | 9 | 6 | 9 | 7 | 9 | 9 | 9 | 9 | 7 | 3 | 7 | 7 | 1 |
| 39 | F | 30588138 | 9 | 9 | 9 | 9 | 9 | 9 | 7 | 9 | 5 | 9 | 9 | 9 | 9 | 9 | 9 | 9 | 9 | 9 | 9 | 1 |
| 40 | F | 29094836 | 8 | 9 | 9 | 9 | 9 | 9 | 7 | 9 | 7 | 9 | 9 | 9 | 9 | 9 | 9 | 9 | 9 | 5 | 9 | 1 |
| 41 | F | 29331846 | 6 | 5 | 6 | 5 | 7 | 7 | 6 | 6 | 5 | 5 | 6 | 7 | 6 | 7 | 7 | 7 | 6 | 7 | 7 | 1 |
| 42 | M | 29325307 | 7 | 7 | 8 | 5 | 6 | 6 | 7 | 7 | 5 | 7 | 7 | 9 | 9 | 9 | 9 | 6 | 7 | 7 | 9 | 1 |
